# Supplementary material for: Exposure to natural hazard events unassociated with policy change for improved disaster risk reduction
Source: Nat Commun. 2021 Jan 8;12:193. doi: 10.1038/s41467-020-20435-2 (PMC7794299; doi:10.1038/s41467-020-20435-2)
Supplement: Supplementary file 1 — Supplementary Information [file 41467_2020_20435_MOESM1_ESM.pdf]

Supplementary information

Exposure to natural hazard events unassociated with policy change for improved disaster risk reduction

Daniel Nohrstedt[1,2,*], Maurizio Mazzoleni[2,3], Charles F. Parker[1,2], Giuliano Di Baldassarre[2,3]

[1]Department of Government, Uppsala University, Uppsala, Sweden. [2]Centre of Natural Hazards and Disaster Science (CNDS), Uppsala University, Uppsala, Sweden. [3]Department of Earth Sciences, Uppsala University, Uppsala, Sweden. [*]e-mail: daniel.nohrstedt@statsvet.uu.se

Contents

1. Supplementary Tables 3

1.1. Supplementary Table 1: Priority for action (PFA) areas and core indicators, Hyogo Framework for Action 2005-2015[1]. 3

1.2. Supplementary Table 2: Correlations between hazard event types and policy change by severity and frequency.. 4

2. Supplementary Figures.. 5

2.1 Supplementary Figure 1. Illustration of baseline periods for normalizing hazard frequency 5

2.2 Supplementary Figure 2. Plots showing the relationship between natural hazard event frequency and severity and normalized aggregated policy change scores 6

2.3 Supplementary Figure 3. Plots showing the relationship between natural hazard event frequency and severity and normalized policy change in relation to different baselines.................................... 7

3. Supplementary results 8

3.1 Case-study validation of PFA change scores: Swaziland.. 8

3.2 Case-study validation of PFA change scores: Chile... 10

4. Supplementary References 12

1. Supplementary Tables

1.1. Supplementary Table 1: Priority for action (PFA) areas and core indicators, Hyogo Framework for Action 2005-2015[1].

**Priority for action 1: Ensure that disaster risk reduction is a national and a local priority with a strong institutional basis for implementation.**
Core indicator 1.1 National policy and legal frameworks for disaster risk reduction exist with decentralised responsibilities and capacities at all levels.<br><br>Core indicator 1.2 Dedicated and adequate resources are available to implement disaster risk reduction plans and activities at all administrative levels.<br><br>Core indicator 1.3 Community participation and decentralisation are ensured through the delegation of authority and resources to local levels.<br><br>Core indicator 1.4 A national multi-sectoral platform for disaster risk reduction is functioning.
**Priority for action 2: Identify, assess and monitor disaster risks and enhance early warning**
Core indicator 2.1 National and local risk assessments based on hazard data and vulnerability information are available and include risk assessments for key sectors.<br><br>Core indicator 2.2 Systems are in place to monitor, archive and disseminate data on key hazards and vulnerabilities.<br><br>Core indicator 2.3 Early warning systems are in place for all major hazards, with outreach to communities.<br><br>Core indicator 2.4 National and local risk assessments take account of regional/trans-boundary risks, with a view to regional cooperation on risk reduction.
**Priority for action 3: Use knowledge, innovation and education to build a culture of safety and resilience at all levels**
Core indicator 3.1 Relevant information on disasters is available and accessible at all levels, to all stakeholders (through networks, development of information sharing systems etc.).<br><br>Core indicator 3.2 School curricula, education material and relevant trainings include disaster risk reduction and recovery concepts and practices.<br><br>Core indicator 3.3 Research methods and tools for multi-risk assessments and cost-benefit analysis are developed and strengthened.<br><br>Core indicator 3.4 Countrywide public awareness strategy exists to stimulate a culture of disaster resilience, with outreach to urban and rural communities.
**Priority for action 4: Reduce the underlying risk factors**
Core indicator 4.1 Disaster risk reduction is an integral objective of environment-related policies and plans, including for land use, natural resource management and adaptation to climate change.<br><br>Core indicator 4.2 Social development policies and plans are being implemented to reduce the vulnerability of populations most at risk.<br><br>Core indicator 4.3 Economic and productive sectorial policies and plans have been implemented to reduce the vulnerability of economic activities.<br><br>Core indicator 4.4 Planning and management of human settlements incorporate disaster risk reduction elements, including enforcement of building codes.<br><br>Core indicator 4.5 Disaster risk reduction measures are integrated into post-disaster recovery and rehabilitation processes.<br><br>Core indicator 4.6 Procedures are in place to assess the disaster risk impacts of major development projects, especially infrastructure.
**Priority for action 5: Strengthen disaster preparedness for effective response at all levels**
Core indicator 5.1 Strong policy, technical and institutional capacities and mechanisms for disaster risk management, with a disaster risk reduction perspective, are in place.<br><br>Core indicator 5.2 Disaster preparedness plans and contingency plans are in place at all administrative levels, and regular training drills and rehearsals are held to test and develop disaster response programmes.<br><br>Core indicator 5.3 Financial reserves and contingency mechanisms are in place to support effective response and recovery when required.<br><br>Core indicator 5.4 Procedures are in place to exchange relevant information during hazard events and disasters, and to undertake post-event reviews.

1.2. Supplementary Table 2: Correlations between hazard event types and policy change by severity and frequency

Income level[a]	All hazard event types		Flood+drought+ storm+landslide+ earthquake		Flood+drought+ storm+landslide		Flood+drought	
	**Slope**	***p*-value**	**Slope**	***p*-value**	**Slope**	***p*-value**	**Slope**	***p*-value**
	Fatalities							
L	-0.26	0.76	-0.27	0.44	-0.28	0.35	-0.23	0.64
LM	-0.02	0.87	0.06	0.54	0.03	0.91	0.22	0.35
UM	-0.09	0.54	-0.19	0.24	-0.26	0.13	-0.20	0.11
H	0.02	0.44	-0.01	0.70	-0.17	0.28	-0.22	0.03*
	Affected people							
L	0.04	0.76	0.06	0.64	0.06	0.64	-0.12	0.94
LM	0.05	0.56	0.05	0.51	0.07	0.47	0.05	0.66
UM	-0.04	0.69	0.02	0.94	-0.002	0.98	-0.08	0.48
H	0.01	0.54	0.01	0.82	0.01	0.82	-0.03	0.59
	Economic losses							
L	0.11	0.73	0.12	0.73	0.18	0.73	0.24	0.60
LM	0.08	0.39	0.08	0.34	0.07	0.39	-0.05	0.66
UM	-0.08	0.18	-0.10	0.11	-0.08	0.17	-0.12	0.14
H	-0.03	0.36	-0.03	0.40	-0.14	0.03*	-0.09	0.04*
	Number of events							
L	-0.87	0.44	-1.19	0.16	-1.12	0.16	-0.20	0.76
LM	-0.13	0.80	-0.18	0.68	-0.20	0.63	-0.18	0.54
UM	-0.37	0.38	-0.39	0.43	-0.27	0.66	0.07	0.81
H	0.29	0.14	0.34	0.05*	0.31	0.02*	0.21	0.1

Supplementary Table 2 | Mann-Kendall trend test (two-sided) and Theil-Sen estimator slope values (Slope) for combinations of natural hazard types in relation to policy change, hazard frequency and severity by income-level.

[a]L=Low-income, LM=Lower-Middle-income, UM=Upper-Middle-income, H=High-income

*indicates significant values

2. Supplementary Figures

2.1 Supplementary Figure 1. Illustration of baseline periods for normalizing hazard frequency

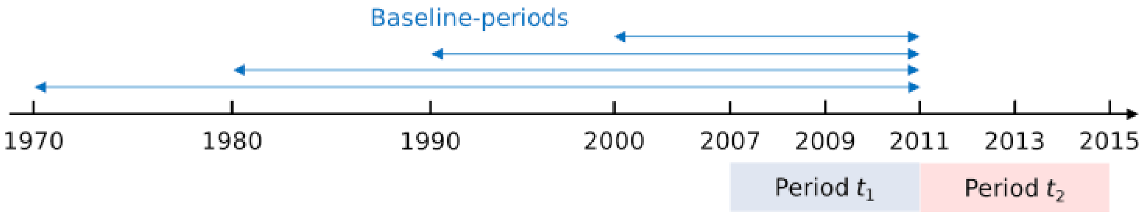

Supplementary Figure 1 | Figure showing four baseline periods for normalizing hazard frequency and severity factors (fatalities, affected people, and economic loss).

2.2 Supplementary Figure 2. Plots showing the relationship between natural hazard event frequency and severity and normalized aggregated policy change scores

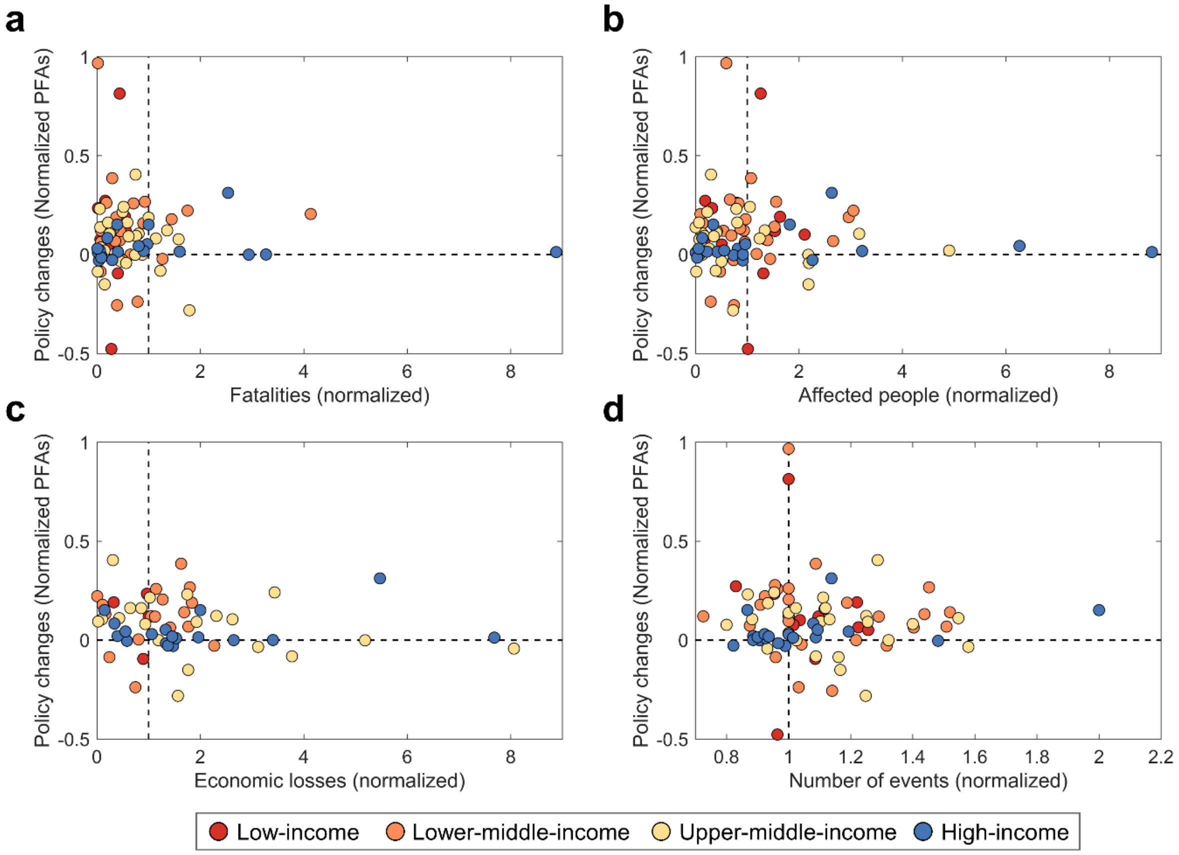

Supplementary Fig. 2| Average policy changes in relation to hazard event frequency and severity measures (normalized against 30yr baseline). Plots showing the relationship between fatalities (a), affected people (b), economic loss (c), number of events (d) and average changes in normalized aggregated PFAs. Source Data are provided as a Source Data file.

2.3 Supplementary Figure 3. Plots showing relationship between natural hazard event frequency and severity and normalized policy change in relation to different baselines

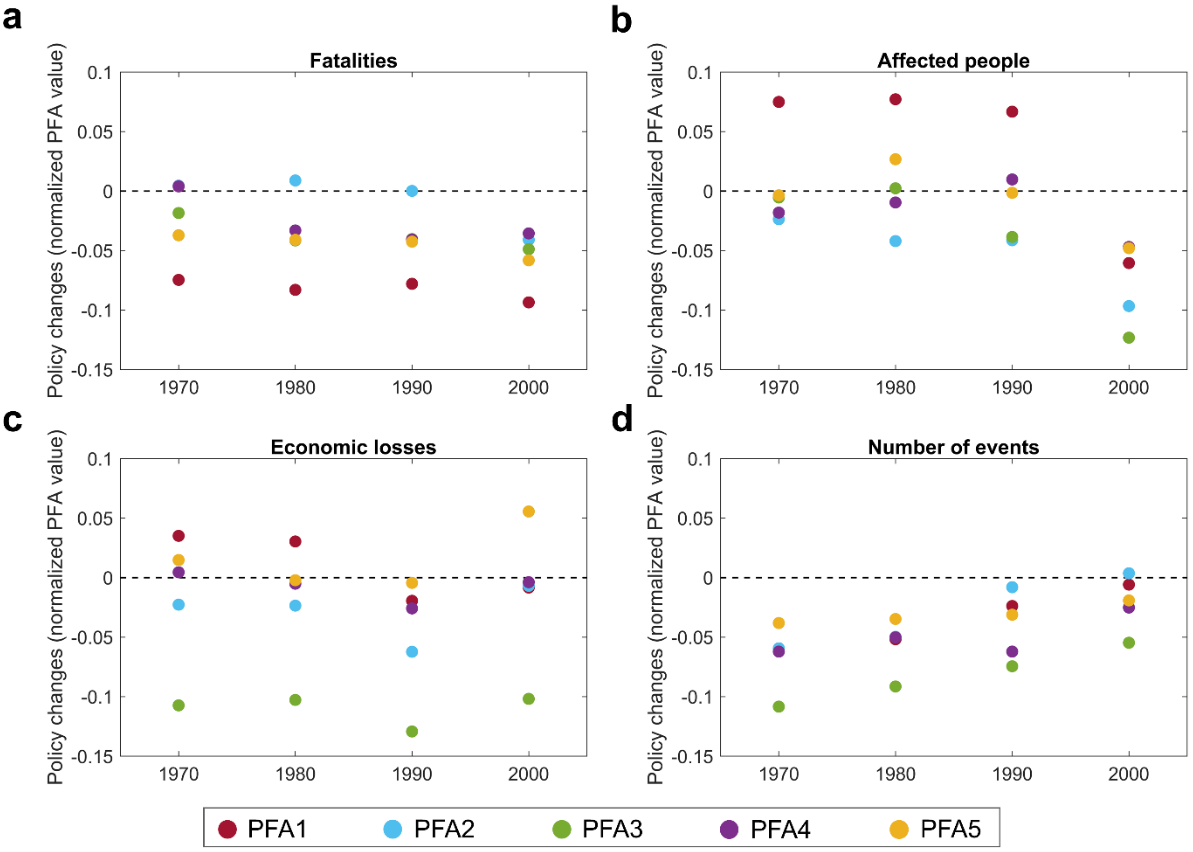

Supplementary Fig. 3 | Plots comparing average normalized values of changes in PFA in case of fatalities (a), affected people (b), economic losses (c), and frequency (d) indices <=1 (hazard events are less or equally frequent and severe as the long-term average, considering the four baseline periods) and indices >1 (events are more frequent and severe than long-term average, considering the four baseline periods). Source Data are provided as a Source Data file.

3. Supplementary results

3.1 Case-study validation of PFA change scores: Swaziland

Swaziland reported the second-highest HFA change scores of all countries included in the dataset, moving from an average score of 1.68 in 2007-2009 to 3.23 in 2013-2015. Below we examine the PFA core indicators that changed the most, from a score of 1 in 2007-2009 to a score of 4 in 2013-2015:

- Core indicator 1.4 A national multi-sectoral platform for disaster risk reduction is functioning.
- Core indicator 3.2 School curricula, education material and relevant training include disaster risk reduction and recovery concepts and practices.
- Core indicator 3.4 Countrywide public awareness strategy exists to stimulate a culture of disaster resilience, with outreach to urban and rural communities.
- Core indicator 4.4 Planning and management of human settlements incorporate disaster risk reduction elements, including enforcement of building codes.

Together the changes reported in these four indicators accounted for 35% of all changes between these two periods.

Swaziland reported it progressed from plans in 2007-2009 to establish a national multi-sector platform (Core-indicator 1.4, progress score = 1, HFA 2007-2009 report p. 5) to implement in 2013-2015 a multi-sector arrangement to coordinate DRR work through the Multi-Hazard Contingency Plan (MHCP). The National Disaster Management Agency (NDMA) assumed the role of overall coordinator of DRR and disaster risk management (DRM) programs, assisted by focal points at government ministries (progress score = 4, HFA 2013-2015 report p. 11-12). Studies[2] confirm that Swaziland made progress during this period in mainstreaming DRR into poverty reduction and national development strategies and also point to the coordinating role of the NDMA[3]. Regarding the timing of implementing a national platform, there is evidence suggesting that this was underway during 2011.[4]

Regarding the integration of DRR in education, Swaziland did not mainstream DRR into education programs in the first HFA evaluation period; however, it did report that a pilot program was underway (Core-indicator 3.2, progress score = 1, HFA 2007-2009 report p. 7). In the HFA evaluation period 2013-2015, Swaziland reported that the National Curriculum Centre had assisted in developing a curriculum that integrated DRR in primary and secondary education programs. A series of workshops was also undertaken to engage DRR practitioners in efforts to develop the DRR curriculum (progress score = 4, HFA 2013-2015 report p.22-23). Some evidence is available concerning the integration of specific DRR aspects into curricula, such as, ecosystems based resilience[5] and a national biosafety framework[6]. Other studies[7] lacked the data to properly document DRR education integration in Swaziland. As a result, the country score concerning PFA core indicator 3.2 could only be partially confirmed by available studies.

The HFA progress reports indicate significant development in Swaziland's strategy towards enhancing public awareness of DRR. In 2007-2009, the country had no formal strategy for public awareness (Core-indicator 3.4, progress score = 1, HFA 2007-2009 report p. 8). Some NGOs assisted communities in developing and implementing preparedness and emergency plans. The 2013-2015 report indicated substantial achievement (progress score = 4, HFA 2013-2015 report p. 26-27) in terms of planning regular national exercises involving DRR stakeholders to build awareness and collaboration among actors. It was also recognized that NGOs remained important for enhancing community preparedness and that the establishment of the NDMA (in 2006) helped to raise awareness. Studies confirm the key role of NGOs in promoting DRR plans and on providing funding for DRR projects in Swaziland.[8] There is evidence to suggest that Swaziland in its policy focus has shifted from response and recovery to DRR, although integration with climate adaptation and natural resource plans still lags behind.[4] The literature also confirms that the NDMA remained important in enhancing coordination in DRR, yet it also suggests that coordination of DRR across policy areas has oscillated between different government bodies.[9]

In 2007-2009, Swaziland reported that due to capacity constraints, DRR had not been effectively incorporated into planning and management of human settlements, although public awareness campaigns had been initiated (Core-indicator 4.4, progress score = 1, HFA 2007-2009 report p. 9-10). The subsequent HFA report for 2013-2015 indicated significant progress (progress score = 4, HFA 2013-2015 report p. 33-35), which was partially attributed to the coordinating role of the National Housing Board (SNHB) and policy changes aiming at clarifying objectives and remaining measures in key areas, such as housing markets and institutional development. Swaziland also reported progress in other areas, including investments in drainage infrastructure in flood-prone areas, slope stabilization in areas with risk for landslides, and provision of housing for low-income households. However, the report also pointed to remaining challenges associated with lack of coordination in land use planning, outdated urban plans, and rapid urbanization. Although most research on planning and management in Swaziland covers periods prior to 2009, some recent work enables comparison with the changes described in the HFA reports. These studies confirm that steps have been taken to integrate DRR with planning and management, including, for example, programs jointly initiated by the government and NGOs to enhance risk mitigation, recovery, and relief.[10,11] There is also evidence pointing to the active role of the SNHB, for example, in promoting the affordable housing mandate. Implementing the mandate, however, has been constrained by the lack of appropriate funding schemes.[12] Another problem has been that many centrally directed projects remain reactive and have neglected the needs of urban households.[10] Studies also confirm what was pointed out in the HFA 2013-2015 report regarding the lack of coordination.[13] Finally, some other evidence describes contests between traditional and urban authorities over planning issues and urban land management.[14]

In summary, the studies reviewed here confirm many of the steps taken by Swaziland from the first (2007-2009) to the fourth (2013-2015) HFA report. This includes measures identified within the four core indicators linked to a national platform (indicator 1.4), education (indicator 3.2), public awareness (indicator 3.4), and planning and management (indicator

4.4). Concerning the scores, the reported changes for these indicators represented a shift from "minor progress" (progress score = 1) to "substantial achievement" (progress score = 4). These changes are largely consistent with what has been reported in the scientific literature, which showed that progress was made in all four indicators. There is also evidence in the literature pointing to a number of remaining challenges, such as lack of coordination in land use planning. The case-study suggests there are a few areas where evidence is sparse (for example, regarding the integration of DRR in education), yet, overall, the studies reviewed here support both the level and content of DRR policy change in Swaziland within the study period.

3.2 Case-study validation of PFA change scores: Chile

Chile had the second-highest PFA change score of all countries in the dataset, as it moved from an average of 2.91 in the first HFA evaluation period to 3.73 in the second period aggregated PFA change score = 0.85). We focus here on the two core indicators with the highest change scores:

- Core indicator 2.1 National and local risk assessments based on hazard data and vulnerability information are available and include risk assessments for key sectors.
- Core indicator 5.3 Financial reserves and contingency mechanisms are in place to support effective response and recovery when required.

Between the first and the second HFA evaluation cycles, Chile's level of progress on core indicator 2.1 changed from 2 to 4, and core indicator 5.3 from 2 to 5. These changes accounted for 28% of Chile's total PFA change between these periods.

In the 2009-2011 HFA progress report (p. 10-11), Chile reported relatively limited progress in conducting risk assessments at local and national levels. Various organizations, including governmental organizations and academic institutions, systematically documented risks associated with natural hazards, particularly geological hazards. However, the information generated by these analyses were not effectively communicated to relevant stakeholders and the general public. According to the HFA report, part of the problem was an ongoing re-organization of the ONEMI (*Oficina Nacional de Emergencia*) Scientific Technical Committee, which was responsible for coordinating and communicating risk analyses by various organizations. Collaboration between government organizations remained challenging, yet the HFA report mentioned that measures were underway to facilitate the exchange of information and more unified communication between organizations. The report also indicated that Chile lacked appropriate technology and monitoring systems. Efforts had been taken to address these issues, for instance, by collaborating with the United States to acquire access to seismic monitoring stations.

In the next report, covering the third HFA evaluation cycle in 2011-2013, Chile reported significant progress in several areas. For instance, it claimed to have taken steps since 2011 to make risk assessment a mandatory component in regional territorial planning. As a result, regional governments now include risk assessments as an instrument in social, economic,

physical, and environmental planning. Through these measures, collaboration between organizations also improved, for instance, by jointly developing guides for how to incorporate risk assessments in regional planning. The HFA report also pointed to remaining challenges. The regional territorial plans (*Planes Regionales Ordenamiento Territorial, PROT*) were voluntary instruments, and hence municipalities were not obliged to apply the guidelines. In addition, there were remaining weaknesses associated with risk assessment methodologies, which made it difficult to assess risks and vulnerabilities in specific territories.

The studies reviewed here confirm the limitations described in the first HFA report as well as the progress depicted in the second HFA report in relation to PFA core indicator 2.1. The literature describes difficulties related to coordinating and effectively interpreting warnings, for example, in relation to the 2010 earthquake and tsunami. Evidence suggests that prior to 2010, risk assessments (tsunami flood maps) were not used in urban policies and planning. After the earthquake, the Chilean government developed a procedure to ensure that risk zones were incorporated into Local Regulation Plans, which was the first time tsunami flood zones were introduced as risk areas in Chile.[15,16] Other research[17] suggests that Chile lacked well-developed plans for updating risk assessments and communicating risk information to stakeholders. Few efforts were undertaken to communicate risk assessments to the general public, which confirms a limitation reported in the first HFA report. Some studies[18] also described a need for a broader risk assessment methodology combining hazard impact with vulnerability assessments of construction and industry, which is consistent with limitations in risk assessment methodologies described in the HFA report. No coordination mechanisms were included in local-level plans to facilitate coordination among government organizations, donor agencies, and other stakeholders.[19] Other studies confirm what was reported in the HFA report concerning remaining challenges to inter-organizational collaboration, which is partially explained with reference to inferior communication technology.[17] The literature also reports that Chile, after the 2010 earthquake, begun to introduce resilience into regional planning, including measures associated with physical, environmental, and social dimensions.[20] It can be noted that most of the studies reviewed here point to the 2010 earthquake and tsunami as a major triggering event leading to a number of changes associated with risk assessment.[21]

Turning to the second PFA core indicator, financial reserves and contingency mechanisms to support disaster response and recovery, the 2009-2011 HFA report (p. 31-32) described limited progress and focused exclusively on funding issues. It argued that although Chile had several funding reserves in place to support disaster response and recovery, the existing system was relatively fragmented and lacked a permanent structure. Disaster funds were distributed across different budget areas. However, a new bill was underway and would create a national civil protection fund to ensure a more stable financial support for disaster prevention and mitigation. The next HFA report (2011-2013) indicated significant progress compared to the previous evaluation cycle, partially due to the establishment of the civil protection fund. It also proposed an increase in the national budget for emergencies to cover costs associated with natural hazards response and recovery measures, which was expressed as a need in the previous report.

Some work suggests that some of the communication issues raised in the first HFA report, particularly the low involvement of the general public in DRR matters, were partially improved in the subsequent period. This was manifested by a more active engagement of neighbourhood and religious organizations in local-level DRR work. It has also been demonstrated that community initiatives received funding and did also influence the revision of municipality plans, programs, and policies.[22] However, there is also evidence suggesting that civil society organizations had limited possibilities to influence public policy associated with community reconstruction.[23] The literature also confirms that the funding streams that have become available in Chile have been important in supporting local DRR work.[24] It also points to different initiatives to reduce financial exposure to future catastrophe losses, which is in line with the initiatives mentioned in the 2011-2013 HFA report.[18]

In summary, studies of DRR work in Chile support what was reported in the HFA evaluation cycles concerning policy changes associated with risk assessments (core indicator 2.1) and funding reserves (core indicator 5.3). These studies confirmed that steps were taken to incorporate risk assessment into regional planning as well as to improve the funding structure that supports disaster response and recovery operations. The available evidence also confirms the level of policy change associated with these measures, from relatively low levels of progress (progress score = 2) to improved commitment and capacity (progress scores = 4 and 5, respectively).

4. Supplementary References

1. United Nations Office for Disaster Risk Reduction (UNISDR). *Implementation of the Hyogo Framework for Action - Summary of reports 2007-2013*. https://www.unisdr.org/we/inform/publications/32916 (2013).

2. Van Niekerk, D. & Wisner, B. Experiences from Sub-Saharan Africa. in *Disaster Management: International Lessons in Risk Reduction, Response and recovery* (eds. López-Carreso, A., Fordham, M., Wisner, B., Kelman, I. & Gaillard, J.) 213–228 (Routledge, 2014).

3. Becker, P., Abrahamsson, M. & Hagelsteen, M. Parallel structures for disaster risk reduction and climate change adaptation in Southern Africa. *Jamba J. Disaster Risk Stud.* **5**, 1–5 (2013).

4. Van Biljon, S. An institutional model for collaborative disaster risk management in the Southern African Development Community. (2014).

5. Lo, V. *Synthesis report on experiences with ecosystem-based approaches to climate change adaptation and disaster risk reduction.* (2016).

6. UN Environment Evaluation Office. *Terminal Evaluation of the UN Environment/Global Environment Facility Project 'Capacity Building for the Implementation of the National Biosafety Framework of Swaziland' Evaluation Office of UN Environment Capacity Building for the Implementation of the National Biosafety Framework of Swaziland UNEP*. (2018).

7. Mutasa, S. & Munsaka, E. Botswana and international policies on the inclusion of disaster risk reduction in the school curriculum: Exploring the missing link. *Int. J. Disaster Risk Reduct.* **40**, 101271 (2019).

8. Van Niekerk, D. Disaster risk governance in Africa: A retrospective assessment of progress in against the hyogo framework for action (2000-2012). *Disaster Prev. Manag.* **24**, 397–416 (2015).

9. Kamara, J., Sahle, B., Agho, K. & Renzaho, A. Governments' Policy Response to Drought in Eswatini and Lesotho: A Systematic Review of the Characteristics, Comprehensiveness, and Quality of Existing Policies to Improve Community Resilience to Drought Hazards. *Discret. Dyn. Nat. Soc.* **26**, 1–17 (2020).

10. Mamba, S. F. Drought, urban resilience and urban food security in kaKhoza, Manzini, Swaziland. *PhD dissertation* (University of the Western Cape, 2019).

11. Mlenga, D. Drought Disaster Risk Reduction through Agriculture Development, the Role of Appropriate Technology Transfer: Experiences from Swaziland. *J. Environ. Earth Sci.* **5**, 6–16 (2015).

12. Nhlabatsi, S. & Dlamini, C. Enabling the Affordable Housing Mandate: The Case of the Swaziland National Housing Board (SNHB). *Am. J. Econ. Financ. Manag.* **1**, 414–420 (2015).

13. Simelane, H. The interplay of urban land tenurial systems and its effects on the poor:A case study of Manzini in Swaziland. (University of Sussex, 2012).

14. Simelane, H. Urban Land Management and its Discontents: A Case Study of the Swaziland Urban Development Project (SUDP). *J. Dev. Stud.* **52**, 797–812 (2016).

15. Herrmann, G. Regulation of Coastal Zones and Natural Disasters: Mitigating the Impact of Tsunamis in Chile Through Urban and Regional Planning. *Issues Leg. Scholarsh.* **11**, 29–44 (2013).

16. Herrmann Lunecke, M. G. Urban planning and tsunami impact mitigation in Chile after February 27, 2010. *Nat. Hazards* **79**, 1591–1620 (2015).

17. Bronfman, N. C., Cisternas, P. C., López-Vázquez, E. & Cifuentes, L. A. Trust and risk perception of natural hazards: implications for risk preparedness in Chile. *Nat. Hazards* **81**, 307–327 (2016).

18. Michel-Kerjan, E., Zelenko, I., Cardenas, V. & Turgel, D. *Catastrophe Financing for Governments*. *OECD Working Papers on Finance* (2011).

19. Rahman, S. & Kausel, T. Coastal Community Resilience to Tsunami: A Study on Planning Capacity and Social Capacity, Dichato, Chile. *J. Humanit. Soc. Sci.* **12**, 55–63 (2013).

20. Villagra, P., Herrmann, G., Quintana, C. & Sepúlveda, R. D. Resilience thinking and urban planning in a coastal environment at risks of tsunamis: the case study of Mehuín, Chile. *Rev. Geogr. Norte Gd.* 63–82 (2016).

21. Platt, S. & So, E. Speed or deliberation: a comparison of post-disaster recovery in Japan, Turkey, and Chile. *Disasters* **41**, 696–727 (2017).

22. Valdivieso Fernández, P. Municipal Governance, Environmental Management and Disaster Risk Reduction in Chile. *Bull. Lat. Am. Res.* **36**, 440–458 (2017).

23. Letelier, F. & Irazábal, C. Contesting TINA: Community Planning Alternatives for Disaster Reconstruction in Chile. *J. Plan. Educ. Res.* **38**, 67–85 (2018).

24. Valdivieso, P., Andersson, K. P. & Villena-Roldán, B. Institutional drivers of adaptation in local government decision-making: evidence from Chile. *Clim. Change* **143**, 157–171 (2017).
