# Supplementary material for: Exposure to natural hazard events unassociated with policy change for improved disaster risk reduction
Source: Nat Commun. 2021 Jan 8;12:193. doi: 10.1038/s41467-020-20435-2 (PMC7794299; doi:10.1038/s41467-020-20435-2)
Supplement: Supplementary file 3 — Reporting Summary [file 41467_2020_20435_MOESM3_ESM.pdf]

nature research

Corresponding author(s): Daniel Nohrstedt

Last updated by author(s): Oct 9, 2020

Reporting Summary

Nature Research wishes to improve the reproducibility of the work that we publish. This form provides structure for consistency and transparency in reporting. For further information on Nature Research policies, see our Editorial Policies and the Editorial Policy Checklist.

Statistics

For all statistical analyses, confirm that the following items are present in the figure legend, table legend, main text, or Methods section.

n/a	Confirmed	
☐	☒	The exact sample size (*n*) for each experimental group/condition, given as a discrete number and unit of measurement
☐	☒	A statement on whether measurements were taken from distinct samples or whether the same sample was measured repeatedly
☐	☒	The statistical test(s) used AND whether they are one- or two-sided<br>*Only common tests should be described solely by name; describe more complex techniques in the Methods section.*
☒	☐	A description of all covariates tested
☒	☐	A description of any assumptions or corrections, such as tests of normality and adjustment for multiple comparisons
☐	☒	A full description of the statistical parameters including central tendency (e.g. means) or other basic estimates (e.g. regression coefficient) AND variation (e.g. standard deviation) or associated estimates of uncertainty (e.g. confidence intervals)
☒	☐	For null hypothesis testing, the test statistic (e.g. *F*, *t*, *r*) with confidence intervals, effect sizes, degrees of freedom and *P* value noted<br>*Give P values as exact values whenever suitable.*
☒	☐	For Bayesian analysis, information on the choice of priors and Markov chain Monte Carlo settings
☒	☐	For hierarchical and complex designs, identification of the appropriate level for tests and full reporting of outcomes
☐	☒	Estimates of effect sizes (e.g. Cohen's *d*, Pearson's *r*), indicating how they were calculated

Our web collection on statistics for biologists contains articles on many of the points above.

Software and code

Policy information about availability of computer code

Data collection	No computer code was used for data collection.
Data analysis	Data analysis was performed by means of code and mathematical algorithm developed using the computer programming language Matlab r2019a. Custom code is deposited in the following public repository: https://www.statsvet.uu.se/research/trampoline/data-repository/

For manuscripts utilizing custom algorithms or software that are central to the research but not yet described in published literature, software must be made available to editors and reviewers. We strongly encourage code deposition in a community repository (e.g. GitHub). See the Nature Research guidelines for submitting code & software for further information.

Data

Policy information about availability of data

All manuscripts must include a data availability statement. This statement should provide the following information, where applicable:

- Accession codes, unique identifiers, or web links for publicly available datasets
- A list of figures that have associated raw data
- A description of any restrictions on data availability

The data that support the findings of this study are available from https://www.preventionweb.net/applications/hfa/qbnhfa/home [disaster risk reduction policy database], https://www.emdat.be/database [EM-DAT database], and https://datahelpdesk.worldbank.org/knowledgebase/articles/906519-world-bank-country-and-lending-groups [World Bank income/levels, fiscal year 2015]. Restrictions apply to the availability of the EM-DAT database, which were used under license for the current study, and so are not publicly available.

Field-specific reporting

Please select the one below that is the best fit for your research. If you are not sure, read the appropriate sections before making your selection.

- [ ] Life sciences
- [x] Behavioural & social sciences
- [ ] Ecological, evolutionary & environmental sciences

For a reference copy of the document with all sections, see nature.com/documents/nr-reporting-summary-flat.pdf

Behavioural & social sciences study design

All studies must disclose on these points even when the disclosure is negative.

Study description	Using quantitative and qualitative data on natural hazard events and disaster risk reduction (DRR) policy, we investigate the relationship between the frequency and severity of natural hazards and changes in national-level DRR policy in 85 countries across the globe.
Research sample	The research sample includes the EM-DAT global dataset of natural hazard events (https://www.emdat.be/), the Hyogo Framework for Action (HFA) disaster risk reduction policy database (https://www.preventionweb.net/applications/hfa/qbnhfa/home), and World Bank data on country income-levels (https://datahelpdesk.worldbank.org/knowledgebase/articles/906519-world-bank-country-and-lending-groups)
Sampling strategy	The sample (n=85 countries) included all countries that submitted at least one HFA report in the first study period (2007-2011) and one in the second study period (2011-2015). We first calculated the difference between the policy scores in between the periods 2007-2011 and 2011-2015. Then, we normalized (i) the total number of hazard events, (ii) natural hazard fatalities, (iii) total people affected, and (iv) economic damage following events occurring between 2011 and 2015 with events occurring during four baseline periods (1970-2011, 1980-2011, 1990-2011 and 2000-2011). A Mann-Kendall trend test (two-sided) was performed between normalized hazard and policy changes by income-levels.
Data collection	Data were collected from the website reported in the previous sections. The study analyzed secondary data sources retrieved from the scientific literature (methods and results in Supplementary information).
Timing	The analyses span the years 1970 and 2015. Four evaluation cycles (2007-2009, 2009-2011, 2011-2013, and 2013-2015) in which countries reported policy changes were considered in relation to natural hazards event data in 1970-2011. Data were collected May 2, 2018 (HFA data), March 20, 2018 (World Bank data), and March 8, 2019 (EM-DAT data).
Data exclusions	Countries (n=65) that participated in the HFA but did not submit reports in both periods were excluded from the study sample.
Non-participation	No participants dropped out or declined participation.
Randomization	Not relevant to the study, which includes all countries participating in the HFA.

Reporting for specific materials, systems and methods

We require information from authors about some types of materials, experimental systems and methods used in many studies. Here, indicate whether each material, system or method listed is relevant to your study. If you are not sure if a list item applies to your research, read the appropriate section before selecting a response.

Materials & experimental systems

n/a	Involved in the study	
x		Antibodies
x		Eukaryotic cell lines
x		Palaeontology and archaeology
x		Animals and other organisms
x		Human research participants
x		Clinical data
x		Dual use research of concern

Methods

n/a	Involved in the study	
x		ChIP-seq
x		Flow cytometry
x		MRI-based neuroimaging
